# Cushing Syndrome as a Manifestation of Neuroendocrine Prostate Cancer: A Rare Presentation Within a Rare Tumor

**DOI:** 10.7759/cureus.18160

**Published:** 2021-09-21

**Authors:** Rute Fernandes, Joana Dos Santos, Frederico Reis, Sofia Monteiro

**Affiliations:** 1 Medical Oncology, Instituto Português de Oncologia do Porto, Porto, PRT; 2 Pathology and Laboratory Medicine, Unidade Local de Saúde de Matosinhos - Hospital de Pedro Hispano, Matosinhos, PRT; 3 Urology, Unidade Local de Saúde de Matosinhos - Hospital de Pedro Hispano, Matosinhos, PRT; 4 Intensive Care Unit, Unidade Local de Saúde de Matosinhos - Hospital de Pedro Hispano, Matosinhos, PRT

**Keywords:** neuroendocrine protate cancer, paraneoplastic syndromes, cushing’s syndrome, neuroendocrine tumors, prostate cancer

## Abstract

Neuroendocrine prostate cancer (NEPC) is a rare entity representing 1% of all prostate malignancies, associated with poor prognosis and often concomitant with paraneoplastic syndromes such as Cushing's syndrome (CS) with ectopic adrenocorticotropic hormone (ACTH) production.

We present a case of a 56-year-old man with recent lower urinary tract symptoms hospitalized for pelvic pain, rectal tenesmus, and fatigue. A CT scan documented a large prostatic mass, adenomegalies, and hepatic lesions. Bone scintigraphy showed dispersed osteoblastic metastization. The patient had uncontrolled hypertension and hypokalemia that lead to the diagnosis of paraneoplastic ACTH-dependent CS. Prostate biopsy confirmed small cell NEPC. Potassium supplementation, anti-hypertensive medication, and metyrapone were initiated. The patient was proposed for palliative chemotherapy but died within a few days from a urinary tract infection.

The authors aim to draw attention to a case of paraneoplastic CS, a rare manifestation, within the rarity that is NEPC.

## Introduction

Prostate cancer (PC) is the second most frequent malignancy in men worldwide. In 2018, 1,276,106 new cases were diagnosed, causing 358,989 deaths (3.8% of all deaths caused by cancer in men) [1,2].

The majority of PC is adenocarcinoma, originating from glandular cells. Neuroendocrine prostate cancer (NEPC) is a rare aggressive variant of PC derived from the neuroendocrine component of the prostate, consisting of a small subset of cells, randomly scattered within the epithelium of the prostate glands [3].

The age-adjusted incidence of NEPC is 0.470 to 0.582/1,000,000 person-years, representing approximately 1% of all prostate malignancies, with five-year cancer-specific survival of 17% [4,5].

The 2016 World Health Organization classification consensus distinguishes adenocarcinoma with neuroendocrine differentiation from well-differentiated neuroendocrine tumors and high-grade neuroendocrine tumors that include small cell neuroendocrine carcinoma (SCC) and large cell neuroendocrine carcinoma [4-6].

Histologic features of prostate SCC include small cells with minimum cytoplasm, fine “salt-and-pepper” chromatin, lack of prominent nucleoli, extensive tumor necrosis, apoptosis, and high mitotic rate. Immunohistochemically, these tumors show chromogranin and synaptophysin positive expression in 61% and 89% of cases, respectively; and 17% of cases are positive for prostate-specific antigen (PSA) and 24% and 35% of cases are positive for p63 and high molecular weight cytokeratin, respectively [7].

Paraneoplastic manifestations - symptoms related to the production of bioactive substances from cancer cells, or occasionally related to immune cross-reactivity to cancer neo-antigens - can occur at any stage [6,7].

Paraneoplastic Cushing’s syndrome (CS) accounts for 10%-20% of CS and develops secondary to neoplastic adrenocorticotropic hormone (ACTH) production, leading to hypercortisolism and its wide range of clinical manifestations including increased mortality. It is mainly derived from the lung, thymus, pancreas, thyroid, chromaffin cell tumors, and rarely from the ovary or prostate [8-10].

## Case presentation

A 56-year-old man presented to the emergency department with pelvic pain, rectal tenesmus, and fatigue. In the past four months, he experienced some episodes of low urinary tract symptoms and initiated treatment for hypertension.

**Figure 1:**
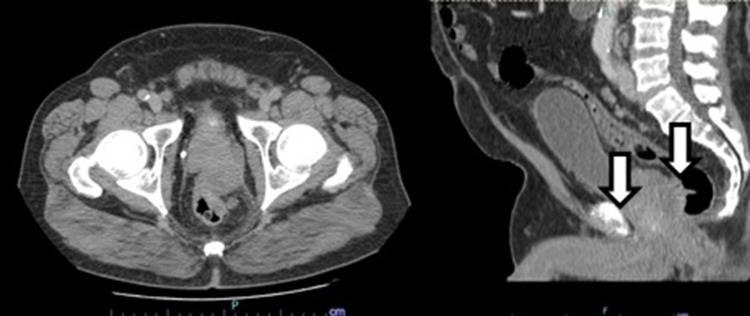
Computed tomography axial plane at pelvic level with large heterogenic prostatic mass (59 x 35 x 33 mm) with loss of cleavage planes with the anterior and left rectum walls.

**Figure 2:**
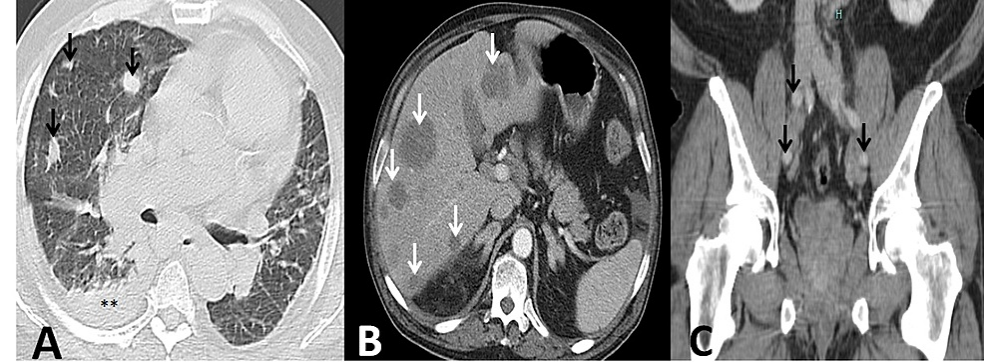
Metastatic disease. A: Diffuse pulmonary lesions. B: Multiple pericentimetric hepatic hypocaptating lesions. C: Pericentimetric iliac adenomegalies (arrows), suggestive of metastatic disease.

Computed tomography (CT) documented a large heterogenic prostatic mass (59 x 35 x 33 mm) with loss of cleavage planes, numerous adenomegalies, multiple pericentimetric hepatic hypocaptating lesions, suggestive of secondary lesions, and evidence of bone involvement (Figures 1, 2).

A urinary catheter was placed due to urinary retention. PSA was elevated with 223 ng/ml (normal <4 ng/ml) and the patient was admitted for pain control and investigation.

At admission, the patient was hypertensive (165/89 mmHg). Laboratory tests evidenced metabolic alkalosis (pH 7.58; HCO3 42 mEq/L), normal sodium of 140 mEq/L, and severe hypokalemia (2.1 mEq/L; normal 3.5-4.5 mEq/L), that showed to be refractory to adequate replacement measures. Hypokalemia investigation showed high levels of morning plasma cortisol (58 𝜇g/L; normal 3.7-19.4 𝜇g/L), high levels of 24 h urinary cortisol (12,333 𝜇g/24 h; normal range 4.3-176) with a negative overnight 8 mg dexamethasone suppression test (81 𝜇g/L; normal <19 𝜇g/L) and elevated levels of plasma ACTH (253 pg/mL; normal 7.2-63.3 pg/mL). A diagnosis of paraneoplastic ACTH-dependent CS was made. The patient was initiated on 250 mg metyrapone daily. The patient's potassium levels increased and 24 h urinary cortisol, after one week on metyrapone, decreased to 3254 𝜇g/24 h. A brain magnetic resonance imaging confirmed the absence of any pituitary abnormality and CT excluded nodules on the thyroid or adrenal gland, confirming ectopic ACTH production.

**Figure 3:**
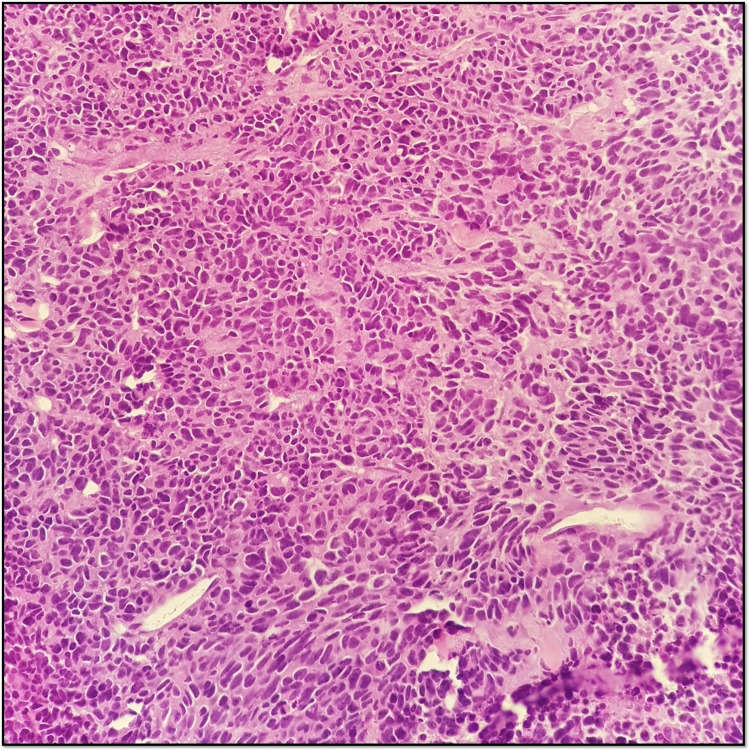
Hematoxylin and eosin staining - small cell neuroendocrine prostate cancer: small cells with minimum cytoplasm and fine “salt-and-pepper” chromatin.

**Figure 4:**
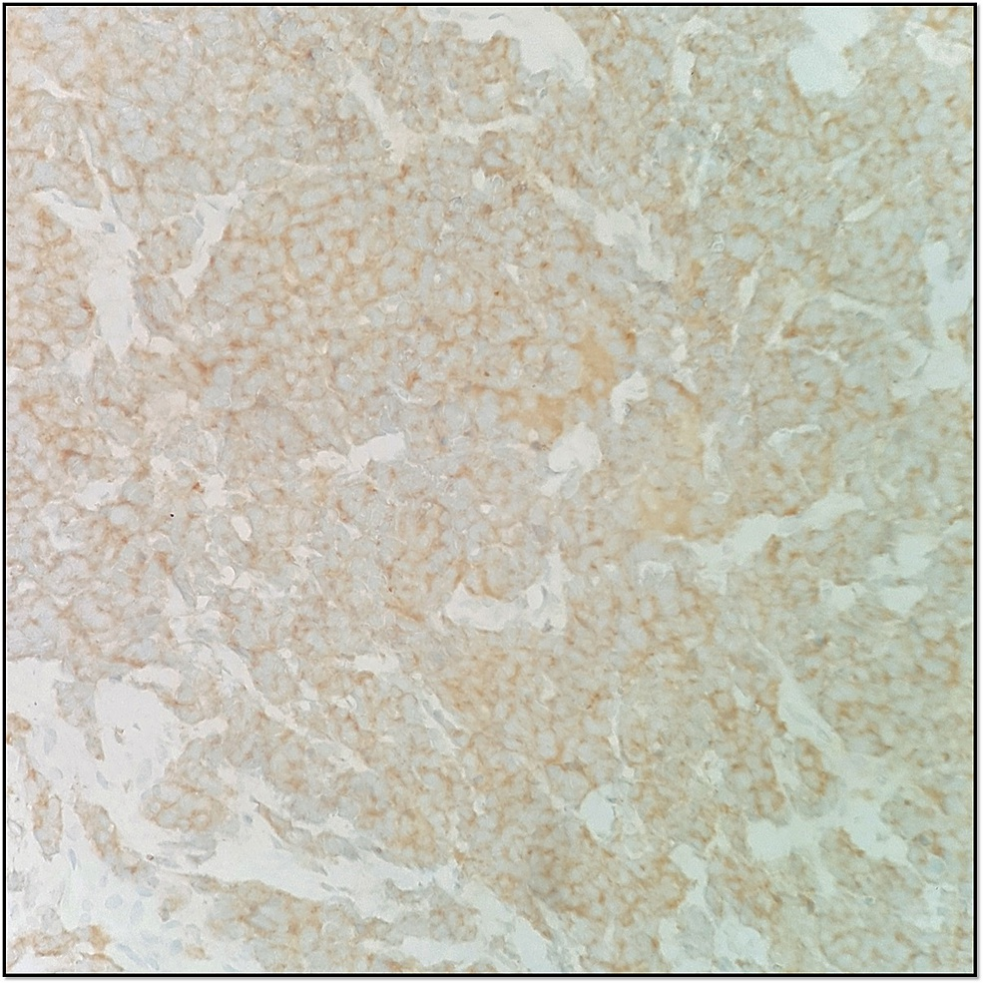
Positive synaptophysin immunohistochemical stain.

**Figure 5:**
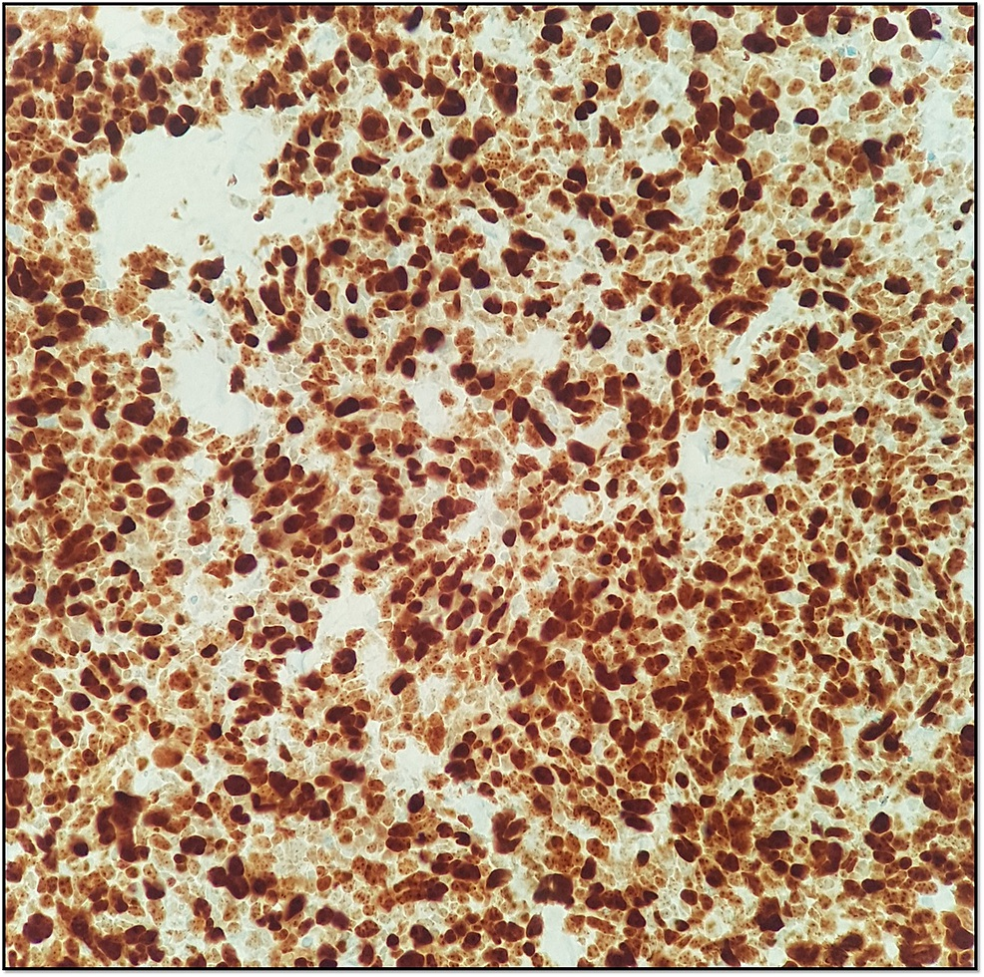
Ki67 immunohistochemical stain - neoplastic cells with high Ki67 expression (90%-100%).

A prostatic biopsy revealed a small cell neuroendocrine carcinoma, positive for synaptophysin and focally for CAM5.2 and PSA, and negative for chromogranin A with a very high Ki67 expression (90%-100%) (Figures 3-5).

Bone scintigraphy showed foci of hyperfixation, suggestive of osteoblastic metastization pattern dispersed along the axial and appendicular skeleton.

Diagnosis of stage IV (cT4N1M1b) NEPC was made. Fluorodeoxyglucose (FDG) positron emission tomography (PET) and 68Ga-DOTA-(Nal3)-octreotide (DOTA-NOC) PET were planned to complete the investigation and palliative chemotherapy was scheduled.

Days after diagnosis, the patient’s condition started to decline and repeated CT scans showed the disease’s progression. The patient developed a nosocomial urinary tract infection with *Escherichia coli *bacteremia that failed to respond to therapy and died within days with multiple organ dysfunctions.

## Discussion

Despite the growing incidence of NEPC in the past two decades, this subtype remains a rare entity. Representing approximately 1% of primary PC, pure de novo SCC is reported to be less than 2% of all prostate malignancies [3-5].

Only 17% of cases have an elevated PSA as seen in our patient, which can mislead the diagnosis to prostate adenocarcinoma. We presented a case of SCC NEPC with ACTH-dependent CS as one presenting manifestation [6,11].

Neuroendocrine tumors are more frequently associated with paraneoplastic syndromes like paraneoplastic CS, which accounts for nearly 20% of all CS manifestations but is rarely associated with NEPC [6,11].

Paraneoplastic CS treatment has a double approach: (1) symptomatic control with drugs promoting cortisol level normalization and (2) tumor burden reduction with chemotherapy, usually consisting of carboplatin or cisplatin plus etoposide, and/or surgery in selected patients [11,12].

Although this case presented with refractory hypertension and hypokalemia, the patient had no further Cushing symptoms such as diabetes or Cushingoid features including thinning of the skin, muscle weakness, weight gain, easy bruising, round face, increased fat around the base of the neck and between the shoulders, or wide purple stretch marks.

The aggressiveness of this tumor, concordant with pathological aspects, was substantiated by marked imaging progression, a key factor for negative outcomes. Moreover, the patient became septic, without response to therapeutic measures.

The patient's outcome is not rare in the NEPC patients. Paraneoplastic CS, especially with extreme hypercortisolism, is very prone to infections. In point of fact, generalized infections represent in this subset of patients one of the main causes of death [6,9].

## Conclusions

Bearing in mind the increasing incidence of NEPC secondary to hormonal therapy, we believe that albeit rare, this form of prostate malignancy, as well as eventual paraneoplastic manifestation, will probably become more frequent throughout the next decades. Quick identification requires high clinical suspicion and prompt management of hypercortisolism, as well as tumor burden reduction therapies.

We aim to raise awareness of a rare manifestation of CS within the rarity that remains NEPC.
